# Coronavirus Disease Pathophysiology: Biomarkers, Potential New Remedies, Comorbidities, Long COVID-19, Post Pandemic Epidemiological Surveillance

**DOI:** 10.3390/ijms241512236

**Published:** 2023-07-31

**Authors:** Jacek Z. Kubiak, Małgorzata Kloc

**Affiliations:** 1 Dynamics and Mechanics of Epithelia Group, Faculty of Medicine, Institute of Genetics and Development of Rennes, University of Rennes, CNRS, UMR 6290, 35000 Rennes, France; 2 Laboratory of Molecular Oncology and Innovative Therapies, Department of Oncology, Military Institute of Medicine, 04-141 Warsaw, Poland; 3 Transplant Immunology, The Houston Methodist Research Institute, Houston, TX 77030, USA; 4 Department of Surgery, The Houston Methodist Hospital, Houston, TX 77030, USA; 5 Department of Genetics, The University of Texas, MD Anderson Cancer Center, Houston, TX 77030, USA

The toughest challenge modern biomedical research ever faced was the rapid understanding of the SARS-CoV-2 physiopathology. Fortunately, this battle against time was successful. The WHO officially declared the end of the pandemic on 5 May 2023. Today, three and half years after the outbreak of the COVID-19 pandemic, and when SARS-CoV-2-induced disease seems to be under control, this deadly disease is one of the best, if not the best, understood human infectious diseases. Our Special Issue participates in this endeavor of enhancing knowledge on SARS-CoV-2’s physiopathology.

According to the WHO record published on 5 July 2023, 767,726,861 COVID-19 cases, 6,948,764 deaths, and 13,461,751,619 vaccine doses were administered worldwide [1]. These numbers illustrate both the profound impact of COVID-19 pandemic on humanity and the rapid and efficient responses from the medical, scientific research, and pharma industries to the threat. Rapid vaccination of a high percentage of the world population delivered immunity against COVID-19 to masses of people. Concomitantly, mutations of SARS-CoV-2 seem associated with reduced severity and participated in the lowered risk to patients’ lives. The complete lack of immune protection at the beginning of the pandemic was the cause of the tragedy we observed from the end of 2019 until 2023.

Despite the progress in understanding, preventing, and treating COVID-19, the focus of biomedical research on this new disease never stopped, even if the danger is much lower than 2–3 years ago. In our Special Issue, we present the whole plethora of such research. In this editorial, we summarize the content of this Special Issue and discuss its impact on the knowledge of COVID-19’s pathophysiology.

During the COVID-19 breakout, when humanity was immune-less against SARS-CoV-2, and there was no proven remedy, many researchers postulated using trained immunity in the fight against the virus [2,3,4]. The review article in our Special Issue by Zapolnik, Kmiecik, Mazur, and Czajka developed this idea further and discussed in detail the potential use of BCG-vaccine-induced trained immunity as a possible tool in the management in COVID-19 [5]. The trained immunity could be a partial solution if vaccines against SARS-CoV-2 appeared inefficient. Fortunately, it was not the case, and the currently used, specific anti-SARS-CoV-2 vaccines eliminated the need for non-specific vaccines. However, the BCG-vaccine-induced trained immunity may still be a solution in future pandemics. One cannot forget, however, about the major limitation of the BCG vaccine, i.e., the presence of a live strain of *Mycobacterium* in it. For this reason, as with other vaccines containing live germs, it can be dangerous for some patients and can participate in spreading the living *Mycobacterium* within the population. This, in turn, could favor mutations, which theoretically could create more dangerous forms of *Mycobacterium*. The review article by the Polish team [5] discussed all the facets of the potential BCG use in this and future pandemics.

The following research papers gathered in our Special Issue show the variety of approaches and results focused on the understanding of the physiopathology of SARS-CoV-2.

Gudowska-Sawczuk et al. [6] focused on the synthesis of kappa (κ) and lambda (λ) Free Light Chains (FLCs) of immunoglobulins in the serums of patients with COVID-19. They argued that elevated levels of FLCs in COVID-19 and healthy patients vaccinated against SARS-CoV-2 reflected hyperactivation of the immune system. Moreover, the correlation they found between FLCs with specific anti-SARS-CoV-2 antibodies and IL-6 strengthened this hypothesis. Thus, the authors claimed that serum levels of FLCs might be used as predictive markers of the course of COVID-19.

Sun and collaborators [7] studied the role of coronavirus nonstructural protein 3 (nsp3), critical for viral replication and host antiviral innate immunity, in infections caused by SARS-CoV-2 and avian coronavirus infectious bronchitis virus (IBV). They showed that nsp3 interacts directly with melanoma differentiation-associated gene 5 (MDA5), inhibiting the MDA5-mediated type I interferon (IFN) response. They confirmed the antagonistic role of nsp3 in the MDA5-mediated type I IFN signaling, which paved a new avenue in the full understanding of the antiviral innate immune response.

The Hutanu et al. group from Romania [8] analyzed the dynamics of the Natural Killer (NK) cells in COVID-19 patients. They focused their attention on different NK subpopulations in relation to different expressions of CD16/CD56. They concluded that the persistency in the circulation of CD56++ NK cells may have important prognostic value in COVID-19 patients with a fatal outcome. Importantly, the total NK cells numbers and the CD16+CD56+ NK subtypes may allow to discriminate between future survivors and non-survivors during the severe COVID-19 progression. These parameters are of great value in COVID-19 management.

The inflammasome, a high molecular weight protein complex activated upon triggering the innate immunity reactions pathway, manifests a cell response to infection and tissue death. The group of Baena Carstens from Brazil [9] analyzed the lung inflammasome activation process in SARS-CoV-2 post-mortem biopsies. They focused on the following inflammasome complex activation markers, TLR4; ACE2; IL-1β; IL-18; NF-κB; ASC; NLRP-3 (or NALP); CASP1; CASP9; GDSM-D; NOX4; and TNF-α, and compared their behavior in patients who died from non-acute pulmonary death causes and those who died of H1N1. They concluded that the inflammasome complex activation was accompanied by activation of the GSDM-D cleavage in COVID-19 patients. This indicated caspase pathways activation and, in turn, pointed to pyroptosis as the important factor in lung injury in COVID-19.

Metabolic alterations induced by SARS-CoV-2 infection are potentially important prognostic biomarkers for COVID-19. The Austrian group of Schuller et al. [10] was interested in identification of such prognostic biomarkers in the end-stage kidney disease (CKD G5) in COVID-19 patients. They analyzed plasma metabolites in COVID-19 patients at different stages with or without hemodialysis (HD). Their targeted metabolomics allowed them to confirm stage-dependent changes in the metabolome in non-HD patients with COVID-19, which were less pronounced in HD patients. Both the increased kynurenine levels and circulating free fatty acids, concomitant with decreased lysophospholipids, allowed them to distinguish patients with moderate COVID-19 from non-infected ones. Kynurenine and lipid alterations also appeared linked to ICAM-1 and IL-15 levels in HD and non-HD patients. Thus, according to the authors, the kynurenine pathway and lipids in the plasma could be considered as universal biomarkers of moderate and severe COVID-19 independent of kidney function.

The article by prof. Fuliński from Cracow, Poland, [11] was much different from other articles in our Special Issue because it dealt with potential physical, and not chemical or biological, means to fight SARS-CoV-2 infection. It was focused on effects of external physical fields on the SARS-CoV-2 ionic E-nanochannel-viroporin, which is vital for the viral cycle and reproduction. The simulations presented in this article demonstrate that external physical fields are able to lower the channel’s currents. The lower the channel’s selectivity, the stronger should be the effects. He showed that the SARS-CoV-2 E-viroporin channel is almost non-selective, which increases the virus vulnerability to external physical fields, in contrast to the much more selective ionic channels present in human cells. Future experimental approaches may result in an innovative method of fighting SARS-CoV-2 and other similar viruses.

The Franco-American group of Celine Boschi [12] studied SARS-CoV-2 Spike protein-induced hemagglutination (HA) of red blood cells (RBCs). They compared the effects of SARS-CoV-2 spike proteins from the Wuhan, Alpha, Delta, and Omicron B.1.1.529 lineages mixed with human RBCs. In all cases, they observed Spike-induced hemagglutination. However, Omicron induced HA at a significantly lower threshold concentration of Spike protein than the remaining SARS-CoV-2 lineages and was highly electropositive in the central protein region. A competitive glycan-binding agent IVM inhibited HA when added to RBCs before the Spike protein and reversed HA when added afterward. Their results showed the potential for the therapeutic use of competitive glycan-binding agents such as IVM and may serve to elucidate rare serious adverse effects caused by COVID-19 mRNA vaccines that use the Spike protein as an antigen.

The group of Anders Larsson [13] studied Shrunken Pore Syndrome (SPS) as a risk marker for COVID-19 patients. SPS is a condition when a selective decrease in the renal filtration of larger molecules is observed. It is frequent in patients during severe COVID-19. It may occur when kidneys are affected by SARS-CoV-2, resulting in acute kidney injury (AKI). They characterized a cohort of COVID-19 patients. In dexamethasone-naïve patients, C-reactive protein (CRP), TNF-alpha, and interleukin-6 were similar between SPS and non-SPS patients. Demographic factors and illness severity were independent predictors of SPS. Also, age and dexamethasone treatment did not impact the frequency of SPS. However, the female gender was associated with a higher proportion of SPS.

Another study on COVID-19 biomarkers involved the article by the group of Dufrusine from Italy [14]. They studied iron Dyshomeostasis and found a functional link to 5-lipoxygenase activation in COVID-19 and in long-COVID-19, possibly via an iron-dependent post-translational mechanism. They also defined other potential markers of the diseases, opening novel opportunities for prevention and treatment.

Pulmonary fibrosis is a common and threatening post-COVID-19 complication involving the plasminogen activator system. This condition was a subject of the study of the Russian group of Shmakova et al. [15]. They compared the data obtained from COVID-19 patients with experimental approaches on urokinase (uPA; encoded by the *PLAU* gene) and urokinase receptor (uPAR; encoded by the *PLAUR* gene) knock-out mice and demonstrated that plasminogen treatment reversed lung fibrosis in Plaur−/− animals. They pointed out that the plasminogen activator system is dysregulated in COVID-19 patients, causing pulmonary fibrosis, and argued that plasminogen could be a treatment for non-resolving post-COVID-19 pulmonary fibrosis.

The American group of Sara Darwish et al. found that COVID-19’s plasma extracellular vesicles increase the density of lipid rafts in small airway epithelial cells [16]. They treated the primary cultures of normal human small airway epithelial cells with EVs from patients and measured the lipid raft formation. They concluded that future studies should investigate whether the increased density of lipid rafts in these cells facilitates viral entry in the recipient cells.

The Spanish group of González-Cuadrado et al. [17], similar to Shuller’s article [10], focused on COVID-19 patients on hemodialysis. They evaluated the immune profile in such patients and found an association with changes in lymphocyte subsets and an increases in pro-inflammatory cytokines and monocyte activation. The worsening observed during the hemodialysis session was accompanied by the augmentation of particular inflammatory cytokines.

Orsolya Dömötör and Éva Enyedy from Hungary [18] characterized plasma protein binding and blood distribution of favipiravir (FAVI), molnupiravir (MOLNU), and imatinib (IMA) used for the treatment of COVID-19. Their goal was to provide a comprehensive overview of these properties.

Alzahrani, Khan, and Ahmad from Saudi Arabia and India [19] analyzed genes potentially implicated in the increased incidence of glioblastoma (inducing significant changes in the immune system) during the COVID-19 pandemic. They delivered a list of genes differentially expressed upon glioblastoma and COVID-19, the profiling of biological processes, the protein–protein interaction network, and the list of selected compounds with high negative correlations for the COVID-19–glioblastoma association with characterized genes.

The research group of Kristina Jevnikar and collaborators from Slovenia [20] presented the comparison of retinal microvasculature in acute COVID-19 and 1 year after hospital discharge with the goal to quantify its possible long-term impairment. They concluded in their work that the transient dilatation of the retinal vessels during acute COVID-19, as well as the retinal nerve fiber layer thickness changes, may be important biomarkers of angiopathy in patients with severe COVID-19.

The Italian group of Maria Denaro et al. [21] presented an epidemiologic study analyzing the course of SARS-CoV-2 genetic variants changes and the differences in the immune responses and clinical symptoms in relation to these variants during the post-pandemic period between 1 January and 31 July 2022 in the Ragusa area in Sicily, Italy. This study showed the full landscape of the characteristics of the disease during the study period in this area.

The study by Susan Costantini et al. from Naples, Italy, [22] identified metabolites and cytokines predictive of outcomes for patients with severe COVID-19 and demonstrated similarities with cancers. They pointed out that the growth factor HGF, lactate, and phenylalanine show a significant prediction of survival.

The group of Stanislav Urazov et al. from Russia and Israel [23] analyzed the levels of interleukin-6 (IL-6) and secretory phospholipase (sPLA2) in the blood samples of patients in relation to the severity and outcome of COVID-19. They showed that the levels of both IL-6 and sPLA2 could be early predictors of aggravation of COVID-19.

The American–Swiss group of Eric Carlson et al. [24] focused on interactions of the SARS-CoV-2 virus with human nicotinic acetylcholine receptors (nAChRs). They showed that the spike protein could interact with nAChRs α4β2 and/or α4α6β2 subtypes, likely at an allosteric binding site. Their results helped to understand the nAChR’s involvement in acute and long-term sequelae associated with COVID-19.

The Bulgarian group of Ekaterina Georgieva [25] analyzed the oxidative stress and albumin-oxidized form related to hypoalbuminemia that reduces treatment effectiveness and increases mortality rate in severe COVID-19 patients. Their final hypothesis was that hypoalbuminemia was not only due to prolonged inflammation but also due to oxidative damage.

Viktor Urbiola-Salvador and collaborators from Poland and Finland [26] described their proteomic study of the blood of patients with COVID-19 focused on identifying biomarkers during the course of COVID-19. They showed that SARS-CoV-2 infection causes distinct plasma protein modifications in the presence of comorbidities despite the interpatient heterogeneity. The key immune signatures found were CD4 and CD28, changing in patients with comorbidities. They identified the increased levels of RBP2 and BST2 proteins with anti-microbial activities and MATN2 and COL6A3 proteins involved in extracellular matrix remodeling, whereas RNF41 was found to be downregulated in patients compared to healthy controls.

The group of Anna Kalinskaya et al. from Russia [27] provided another proteomic study of blood plasma of post-COVID-19 patients with Acute Myocardial Infarction (AMI), which is a cardiovascular complication after SARS-CoV-2 infection. The comparison of AMI control (without COVID-19) and AMI post-COVID patients showed diminished levels of proteins related to inflammation and the hemostasis-like lipopolysaccharide-binding protein, C4b-binding protein α-chain, plasma protease C1 inhibitor, fibrinogen β-chain, vitamin K-dependent protein S, and altered correlations between inflammation and fibrinolysis. The important new finding was that the AMI post-COVID patients had a lower increase in acute-phase proteins and hemostatic markers. The authors argued that it could be related to the prolonged immune system alterations after COVID-19. 

In summary, most of the articles in this Special Issue are devoted to studies of novel biomarkers of COVID-19 outcomes, better understandings of SARS-CoV-2 infections, and research into preventive means. The readers may judge for themselves if the articles gathered in our Special Issue substantially increase our knowledge of COVID-19.
